# Annexin-A5 organized in 2D-network at the plasmalemma eases human trophoblast fusion

**DOI:** 10.1038/srep42173

**Published:** 2017-02-08

**Authors:** Severine A. Degrelle, Pascale Gerbaud, Ludovic Leconte, Fatima Ferreira, Guillaume Pidoux

**Affiliations:** 1 INSERM, U767, Cell fusion, Paris, F-75006 France; 2 Université Paris Descartes, Paris, F-75006 France; 3 PremUp, Paris, F-75006 France; 4 UMR-S1180, Inserm, Univ. Paris-Sud, Université Paris-Saclay, Châtenay-Malabry, France; 5 UMR144, Institut Curie/CNRS, Cell and Tissue Imaging Platform, Paris, France

## Abstract

Only a limited number of human cells can fuse to form a multinucleated syncytium. Cell fusion occurs as part of the differentiation of some cell types, including myotubes in muscle and osteoclasts in remodeling bone. In the differentiation of the human placenta, mononuclear cytotrophoblasts aggregate and fuse to form endocrinologically active, non-proliferative, multinucleated syncytia. These syncytia allow the exchange of nutrients and gases between the maternal and fetal circulation. Alteration of syncytial formation during pregnancy affects fetal growth and the outcome of the pregnancy. Here, we demonstrate the role of annexin A5 (AnxA5) in syncytial formation by cellular delivery of recombinant AnxA5 and RNA interference. By a variety of co-immunoprecipitation, immunolocalization and proximity experiments, we show that a pool of AnxA5 organizes at the inner-leaflet of the plasma membrane in the vicinity of a molecular complex that includes E-Cadherin, α-Catenin and β-Catenin, three proteins previously shown to form adherens junctions implicated in cell fusion. A combination of knockdown and reconstitution experiments with AnxA5, with or without the ability to self-assemble in 2D-arrays, demonstrate that this AnxA5 2D-network mediates E-Cadherin mobility in the plasmalemma that triggers human trophoblasts aggregation and thereby cell fusion.

The cell fusion process consists of the formation of multinucleated syncytia by the mixing of cellular membrane components and cell contents from two or more cells. This complex phenomenon occurs in fertilization, placentation, fetal development, skeletal muscle formation and bone homeostasis^1^,^2^,^3^,^4^. Cell fusion processes consist of three distinct stages^5^, the competence, commitment and full fusion stage. The competence stage is characterized by the loss of cellular proliferation and the differentiation into fusion-competent cells. This includes cell migration, morphological changes and secretion or response to extracellular signals such as growth factors, cytokines and hormones^5^. The commitment stage describes the recognition of fusion partners, followed by the cellular adhesion and inter-cellular communication. This leads to activation, expression or assembly of the fusogenic machinery and to the synchronization of fusion-competent cells through the exchange of fusogenic signals. These two first stages are a prerequisite to promote the cell fusion with fusion pore formation between aggregated cells and the mixing of cellular content^6^. Several proteins, protein macrocomplexes and cellular signaling pathways have been reported to trigger trophoblast fusion^5^. Tight junction (*e.g.* ZO-1), adherens junction (*e.g.* cadherins) and gap junction (*e.g.* connexins) proteins have been shown to play a fundamental role during the commitment stage of trophoblast fusion^5^. E-cadherin is a transmembrane protein that mediates mononuclear cell aggregation and adherens junction formation between fusion-competent cells essential for cell fusion^7^. The E-cadherin extracellular N-terminal domain generates cellular adhesion by clustering with homotypic and heretotypic cadherins through the neighboring cell. This cellular adhesion stabilizes the cell membrane and allows polarization to the future fusion area. This triggers the clustering of fusogenic proteins or proteins initiating trophoblast fusion at the right time and the right place to the plasma membrane^5^. Gap junctions are responsible for communication between adjacent cells and are composed of connexins. Gap junction channels allow the exchange of small molecules, second messengers and fusogenic signals facilitating cellular coordination, spatial compartmentalization and myoblast or trophoblast fusion^8^,^9^. Finally, syncytins trigger lipid mixing and fusion pore formation in placentation, fertilization, myoblast and osteoclast fusion^7^.

Human embryo implantation requires placentation, a process in which fetal trophoblasts in early pregnancy invade the maternal endometrium. Two specific pathways of trophoblast differentiation characterize human placental development. Extravillous cytotrophoblasts display an invasive nature and play an essential role in anchoring chorionic villi^10^. While villous cytotrophoblasts fuse throughout pregnancy to form multinucleated syncytia on chorionic villi that extends into the maternal placental blood circulation to form an interphase allowing effective exchange of gases and nutrients in the intervillous chamber^11^. Moreover, these multinucleated syncytia produce and secrete pregnancy-specific hormones^12^. It is noteworthy that feto-maternal exchanges and hormonal functions are necessary for fetal growth and outcome of the pregnancy. The *in vivo* fusion process of the human placenta is reproducible *in vitro* using purified cytotrophoblasts, which aggregate and then fuse to form non-proliferative, multinucleated, endocrinologically active syncytiotrophoblasts^13^. The cytotrophoblast plays an essential role during human pregnancy, through its ability to differentiate into syncytia. Abnormal cytotrophoblast differentiation and cell fusion have severe consequences on fetal growth and pregnancy outcome. These are characterized by both a decrease in chorionic villus volume and surface area, which are severely compromised in intrauterine growth restriction (IUGR) and preeclampsia^14^.

Annexins (AnxA) are members of a soluble protein family, in humans composed of 12 members that bind to membranes exposing negatively charged phospholipids in a Ca^2+^ dependent manner. AnxA consist of an annexin core and a variable amino (Nt)-terminus domain. The annexin core displays Ca^2+^-binding sites, which mediate AnxA binding to membranes^15^. Membrane binding depends on the content of negatively charged phospholipids (such as phosphatidylserine (PS)) and the Ca^2+^ concentration^15^,^16^. The Nt domain confers the functional specificity of annexins^16^. AnxA have been described to be involved in numerous membrane-related processes (*e.g.* exo- and endocytosis, vesicle trafficking, membrane aggregation, fusion and cell membrane repair)^15^,^17^. Annexin A5 (AnxA5) is the smallest AnxA and contains only the annexin core. Interestingly, AnxA5 displays a structural property to self-organize into a two dimensional (2D) array upon binding to biological membrane. A rise in Ca^2+^ concentration triggers binding of monomeric AnxA5 molecules to the membrane surface containing negatively charged phospholipids, which subsequently first assemble rapidly into trimers and then self-organize into 2D ordered arrays, even at low surface density^18^. Recently, it has been reported that AnxA1 and AnxA5 are important for sequential steps of myoblast fusion^19^,^20^. Furthermore, it is noteworthy that AnxA5 2D-arrays controls trophoblast cell membrane repair^21^. Here, we show that a pool of AnxA5 clusters at the plasma membrane subset of aggregated trophoblasts, in the vicinity with E-cadherin, α-catenin and β-catenin. Finally, we assign a new function to AnxA5 by showing that it is involved in the regulation of trophoblast fusion by self-organizing in 2D-arrays at the inner-leaflet, which facilitates E-cadherin plasma membrane mobility and cellular aggregation necessary to initiate trophoblast fusion.

## Results

### AnxA5 localizes at the cellular plasma membrane of trophoblasts

**Figure 1:**
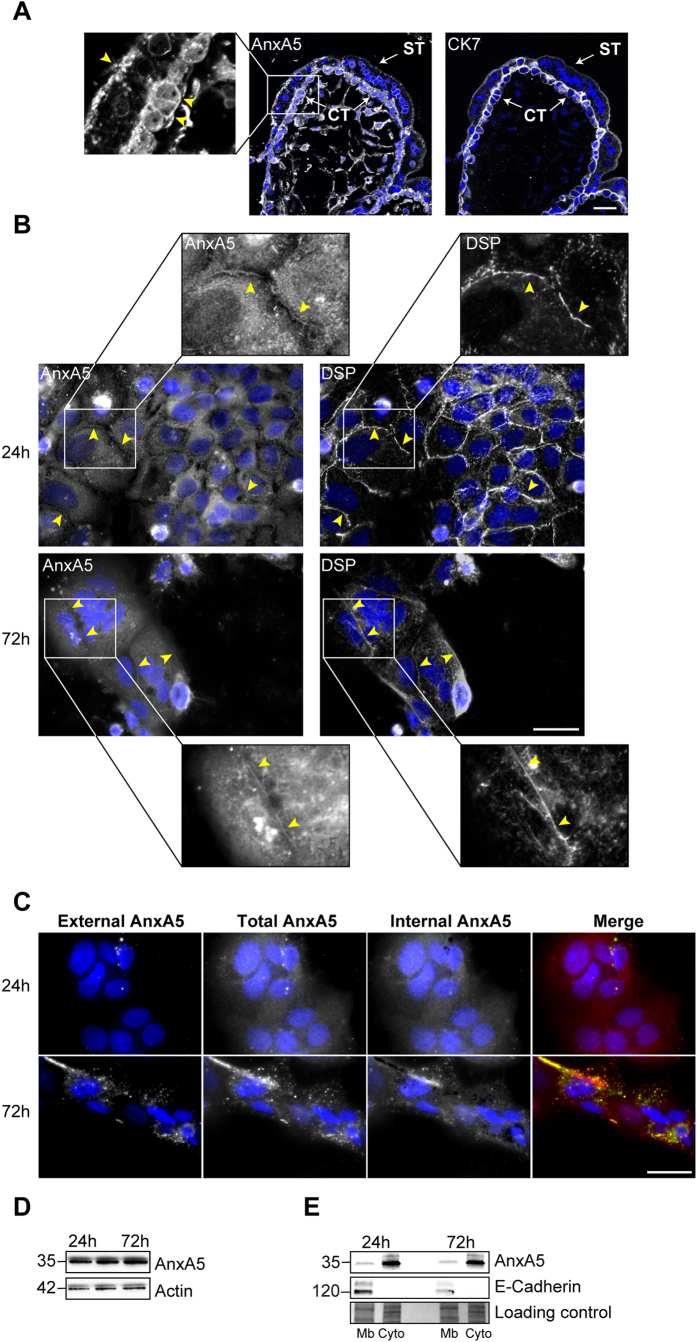
AnxA5 is expressed at the junction between cyto- and syncytiotrophoblasts. (**A**) Immunostaining of AnxA5, CK7 and nuclei (TOPRO-3) in human placental biopsies. The magnified views show representative areas of AnxA5 localization (yellow arrowheads). CT, cytotrophoblast; ST, syncytiotrophoblast. Scale bar 10 μm. (**B**) Immunostaining of AnxA5, desmoplakin (DSP) and nuclei in primary human trophoblasts at 24 h (upper panel) and 72 h (lower panel) of culture. The magnified views show representative areas of AnxA5 and DSP localization (arrowheads). Scale bar: 15 μm. (**C**) Differential labelling of cell-surface (left column; external AnxA5) and total AnxA5 (middle left column) in primary human trophoblasts at 24 h (upper row) and 7 h (lower row) of culture. Internal AnxA5 (middle right column) was obtained after the removal of external AnxA5 from total AnxA5 staining. Merge consists of the association of external and total AnxA5 staining (right column). Nuclei were counterstained in blue with DAPI. Scale bar: 15 μm. (**D**) Immunoblots of AnxA5 and actin levels in trophoblasts at 24, 48 and 72 h of culture. (**E**) Immunoblots of AnxA5 and E-Cadherin in cellular membrane (Mb) and cytosolic (Cyto) fractions from trophoblasts after 24 and 72 h of culture. Protein staining is shown as loading control (bottom panel).

Of all human organs, the human placenta is known to have the highest AnxA5 protein expression. In trophoblasts AnxA5 represents approximately 1% of the total protein level (Supplementary material Fig. S1A). Recently, AnxA5 has been shown to play a role in myoblast fusion^20^. We therefore studied the AnxA5 subcellular localization in human first trimester placenta biopsies by co-immunostaining of AnxA5 and cytokeratine 7 (CK7), a specific marker of trophoblasts (Fig. 1A). We noticed the location of AnxA5 at the surface of the villi in the syncytiotrophoblast but also at the plasma membrane subset of the mononucleated cytotrophoblasts and at the junction with syncytia, where cell fusion occurs (Fig. 1A, yellow arrowheads in magnified views). We observed no immunostaining with isotype-matched IgG control (Supplementary material Fig. S1B). Furthermore, AnxA5 subcellular localization was examined by co-immunostaining of AnxA5 and desmoplakin (DSP, a cellular membrane protein component of desmosomes) in primary human trophoblasts (Fig. 1B). AnxA5 immunostaining revealed a perinuclear and a cytoplasmic localization as well as a distribution that overlaps with the plasma membrane at the junction between cytotrophoblasts or syncytiotrophoblasts (Fig. 1B, yellow arrowheads in magnified views). To decipher from which side of the cellular membrane the AnxA5 localized, a differential labelling of cell-surface and total AnxA5 was performed and revealed by immunostaining (Fig. 1C). Interestingly, a weak or no AnxA5 staining was observed at the outer membrane of cytotrophoblasts as compared with the intracellular AnxA5 staining (Fig. 1C, upper row). Intracellular AnxA5 staining was obtained after subtraction of external to total AnxA5 staining (Fig. 1C, middle right column). Interestingly, after fusion, syncytiotrophoblasts showed AnxA5 externalization (Fig. 1C, lower row). These indicate that an exposition of AnxA5 at the outer membrane subset appeared during trophoblast fusion after the commitment stage. No staining was observed with isotype-matched IgG controls (Supplementary material Fig. S1C). Finally, the level of AnxA5 was examined by immunoblot in primary human trophoblast extracts and cell membrane or cytosolic fractions during cell fusion. As evident from immunoblots, the level of AnxA5 protein expression remained constant during trophoblast fusion (Fig. 1D,E). The expression of AnxA5 protein examined after cell fractionation was found at the membrane subset and in a greater extends in the cytosolic compartment (Fig. 1E). This supports AnxA5 subcellular localization observed in our immunocytofluorescence studies (Fig. 1B,C). As expected, E-cadherin protein expression showed an exclusive expression at the cell membrane subset of human trophoblasts (Fig. 1E).

### Recombinant AnxA5 induces trophoblast fusion with PS flip

**Figure 2:**
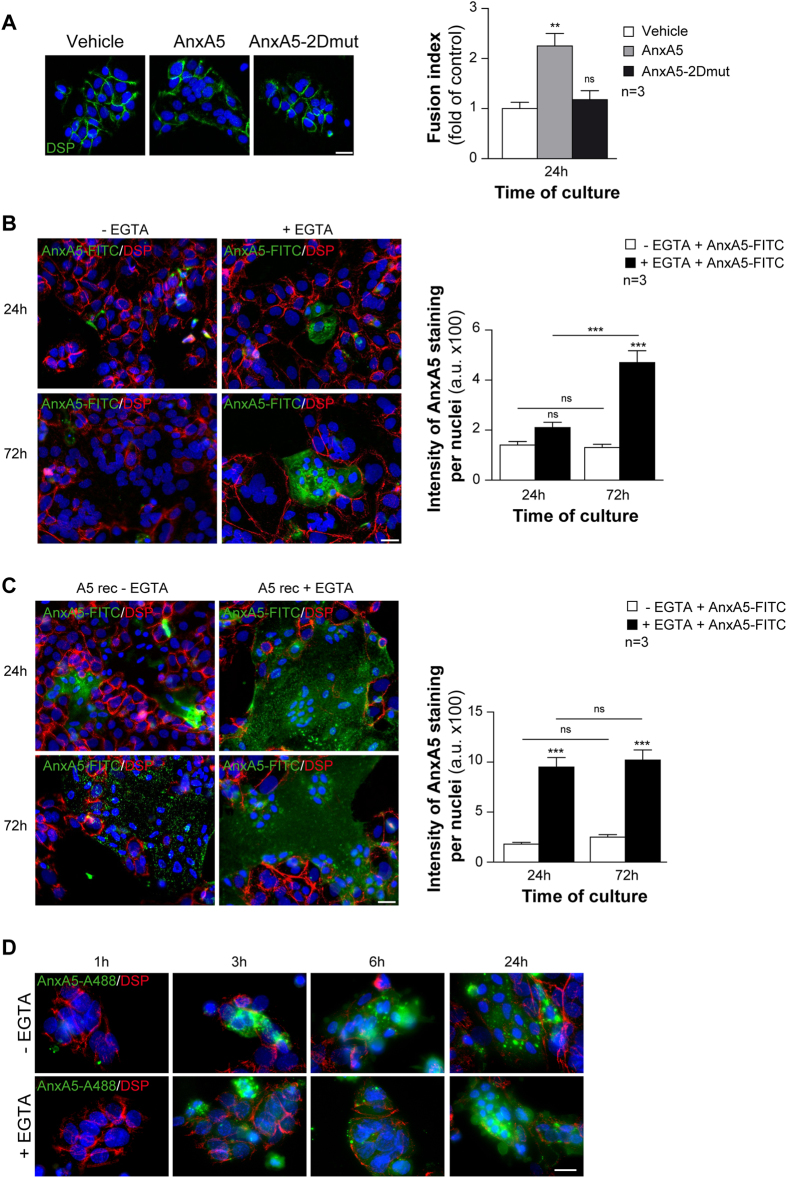
Recombinant AnxA5 induces human trophoblast fusion and PS flip. (**A**) Trophoblasts were first incubated with recombinant AnxA5 or AnxA5-2Dmut and immunostained for desmoplakin (DSP, green). Nuclei were counterstained with DAPI (left panel). Effects of recombinant AnxA5 and AnxA5-2Dmut incubation (10 μg each) on cell fusion assessed as fusion indices (right panel). (**B**) To quantify phosphatidylserine (PS) flip, trophoblasts were treated with or without EGTA and incubated with recombinant AnxA5-FITC. Trophoblasts were next immunostained for desmoplakin (DSP, red) and nuclei were counterstained with DAPI at 24 or 72 h of culture (left rows). PS flip analysis was assessed as AnxA5 fluorescence intensity bound to trophoblasts (right histograms). (**C**) Trophoblasts were incubated with recombinant AnxA5 (10 μg/ml) for 24 or 72 h and subsequently treated with or without EGTA followed by AnxA5-FITC incubation. Trophoblasts were next immunostained for desmoplakin (DSP, red) and nuclei were counterstained with DAPI at 24 or 72 h of culture (left rows). PS flip analysis was assessed as AnxA5 fluorescence intensity bound to trophoblasts (right histograms). (**D**) Subcellular localization of AnxA5 that induced trophoblast fusion was monitored over the time after cell incubation with AnxA5-A488 (10 μg/ml) in presence or absence of EGTA treatment. Next, trophoblasts were immunostained for desmoplakin (DSP, red) and nuclei with DAPI (left panel). Scale bar: 15 μm. Quantitative results are expressed as the mean ± SEM. (n** = **3 independent experiments); **p < 0.01; ***p < 0.001; ns, non-significant.

To assess the role of AnxA5 in human trophoblast fusion, cells were cultured for 24 h in absence or presence of recombinant AnxA5 wild type or a mutant AnxA5 unable to self-assemble into 2D-arrays (which has substitution R18E, R25E, K29E, K58E and K193E to abolish AnxA5 2D-arrays formation; AnxA5-2Dmut)^22^. Subsequently, cell fusion was characterized by fusion assay. Mononuclear cytotrophoblasts in culture spontaneously aggregated after 24 h as evident from desmoplakin immunostaining (Fig. 2A). By contrast, trophoblasts treated with recombinant AnxA5 presented discontinuous desmoplakin immunostaining already after 24 h of culture, indicating the presence of syncytia (Fig. 2A). AnxA5 triggered cytotrophoblasts fusion in a concentration dependent manner (Supplementary material Fig. S1D). Interestingly, cells cultured with AnxA5-2Dmut behaved like control cultures, which suggests that AnxA5 organized in a 2D-arrays drives the fusion process (Fig. 2A). In a search for exposed PS, cells were subjected to an AnxA5-FITC binding assay. Cells were treated with EGTA, which chelate calcium and dissociate putative preexisting AnxA5 bound to exposed-PS (Fig. 2B). It is noteworthy that we did not observe significant changes in trophoblast fusion after EGTA treatment (Supplementary material Fig. S1E). After 24 h of culture, we noticed that a few PSs were exposed by cytotrophoblasts as evident from weak AnxA5-FITC staining and corresponding histograms from control and EGTA-treated cells (Fig. 2B). This suggests that few cytotrophoblasts exposed PS. In contrast, at 72 h of culture, EGTA-treated syncytiotrophoblasts revealed a large exposition of PS that was masked in untreated cells by endogenous AnxA5 (Fig. 2B; p < 0.001). These indicate that trophoblast fusion is associated with an increase in PS exposition masked by endogenous AnxA5. These data are in agreement with our differential labelling experiments (Fig. 1C). Subsequently, trophoblast cultures were induced for fusion with recombinant AnxA5 (10 μg/ml) and subjected to AnxA5-FITC binding assay (Fig. 2C). As evident from histograms and immunostaining, recombinant AnxA5 induced syncytia formation with a large exposition of PS masked by AnxA5 (Fig. 2C; p < 0.001). It is noteworthy that AnxA5 bound to exposed-PS is often associated with early apoptosis. Thus, the level of apoptosis was evaluated during cell fusion with or without addition of recombinant AnxA5 (10 μg/ml) by quantifying the level of immuno-positive cells for cleaved cytokeratin 18 (cCK18; early marker of apoptosis) (Supplementary material Fig. S2A,C). As evident from histograms no significant changes in trophoblast viability during cell fusion were observed in presence or absence of recombinant AnxA5. Moreover, correlation between apoptosis and PS exposure was investigated by searching for double positive cCK18 and AnxA5-FITC stained cells (Supplementary material Fig. S2C,D). We noticed a weak or a partial overlap between apoptotic trophoblast and PS exposure during the fusion process with or without AnxA5 pre-incubation, suggesting for an absence of correlation between PS flip and trophoblasts apoptosis (Supplementary material Fig. S2C,D; Pearson’s R coefficient ranged from 0.17 to 0.65). Finally, to monitor the subcellular localization of exogenous AnxA5, trophoblasts were incubated with AnxA5-A488 in a time course experiment. Trophoblasts were washed with or without EGTA and next subjected to desmoplakin immunostaining (Fig. 2D). After 1 h of incubation with AnxA5-A488 none of the trophoblasts were stained. Whereas, we noticed in both conditions presence of aggregated green cells after 3 h and green syncytia after 6 h incubation. Larger green syncytia were observed in both conditions after 24 h incubation with AnxA5-A488. All together, these indicate that exogenous AnxA5 was quickly internalized and induced fusion between 3 and 6 h post incubation.

### AnxA5 2D-network is required for human trophoblasts fusion

**Figure 3:**
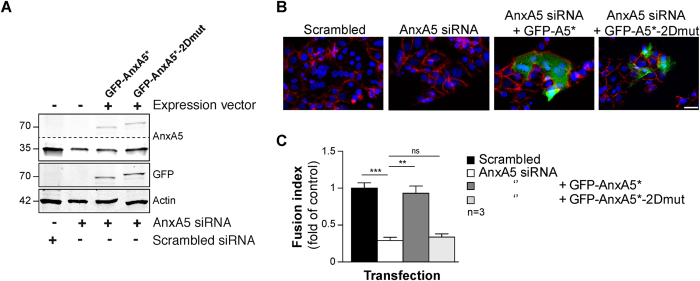
Endogenous AnxA5 organized in 2D-network facilitates cell fusion. (**A**) Trophoblast were transfected with AnxA5 siRNA or scrambled control alone or together with mammalian expression vectors directing the expression of siRNA-resistant GFP-AnxA5 (GFP-AnxA5*) or GFP-AnxA5 with mutations in 2D-array organization sites (GFP-AnxA5*-2Dmut) and subjected to immunoblot analysis with the indicated antibodies. Dotted lines indicate parts combined from a single gel and exposure. (**B**) Cells with AnxA5 knockdown and/or reconstitution as in A were stained for desmoplakin (red) and nuclei (DAPI, blue). Scale bar: 15 μm. (**C**) Graphs represent fusion indices at 72 h of culture treated as in (**A** and **B**). Quantitative results are expressed as the mean ± SEM. (n** = **3 independent experiments); **p < 0.01; ***p < 0.001.

We demonstrated that exogenous AnxA5 accelerated the process of fusion and next, we tested the hypothesis that the trophoblast fusion is facilitated through endogenous AnxA5 organized in a 2D network. We performed cell fusion assays on trophoblasts depleted of endogenous AnxA5 by siRNA transfection. To rescue AnxA5 knockdown, mammalian expression vectors were introduced encoding AnxA5 fused to GFP and made insensitive to functional siRNA (GFP-AnxA5*), with or without substitutions that abolish AnxA5-2D network formation (GFP-AnxA5*-2Dmut) (Fig. 3). RNA interference-mediated knockdown of AnxA5 reduced protein expression by approximately 80% after normalization to actin levels and as compared to cells transfected with scrambled siRNA (Fig. 3A and Supplementary material Fig. S3A; p < 0.001). As evident from microscopy pictures and fusion index (Fig. 3B,C and Supplementary material Fig. S3B), AnxA5 knockdown decreased trophoblast fusion compared to cells transfected with scrambled control (p < 0.001). However, cells reconstituted with GFP-AnxA5* after knockdown of endogenous AnxA5, did form syncytia (Fig. 3B,C). By contrast, cytotrophoblasts reconstituted with GFP-AnxA5*-2Dmut did not fuse (Fig. 3B,C). Furthermore, we employed a strategy of RNA interference and reconstitution with addition of recombinant AnxA5 in culture media (Supplementary material Fig. S3B). Similarly to reconstitution experiments with GFP-AnxA5*, cells in which endogenous AnxA5 was knocked down formed syncytia after incubation with recombinant AnxA5 (Supplementary material Fig. S3B). Our experiments suggest that endogenous AnxA5 is necessary for human trophoblast fusion and the formed 2D-network plays a key role in the regulation of this process.

To monitor the kinetics of AnxA5 translocation to the plasma membrane, trophoblasts were transfected with GFP-AnxA5*, GFP-AnxA5*-2Dmut or mCherry-AnxA5* and mCherry-AnxA5*-2Dmut and incubated with ionomycin to raise the intracellular concentration of calcium (Supplementary material Fig. S3C,D). The calcium fluxes induced by ionomycin led to a rapid and progressive translocation of tagged-AnxA5 to the plasma membrane, vesicles and perinuclear area (Supplementary material Fig. S3C,D). Interestingly, we previously observed these locations with endogenous AnxA5 in human trophoblasts (Fig. 1B). Next, we monitored the spontaneous calcium waves in primary human trophoblasts cultured with Fluo4-AM (Supplementary material Fig. S3E). Kymograms highlighted for an irregular activity of calcium waves in primary human trophoblasts (Supplementary material Fig. S3E). Taken together, these spontaneous rises in intracellular calcium could trigger endogenous AnxA5 translocation and location at the plasmalemma to facilitate trophoblast fusion.

### Identification of AnxA5-binding proteins at the cellular membrane subset of trophoblasts

**Figure 4:**
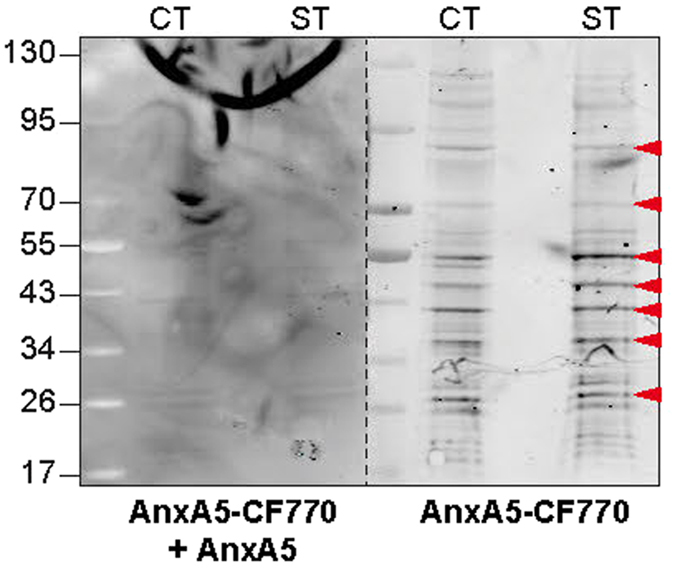
AnxA5 interactome at plasmalemma of human trophoblast. Proteins purified by sub-cellular membrane fractionation from cytrophoblasts (CT) or syncytiotrophoblasts (ST) were subjected to a solid phase binding assay-using AnxA5-CF770 (0.5 μM; AnxA5-overlay) as a probe in the absence or presence of an excess of unconjugated AnxA5 (1 μM). Red arrowheads indicate putative interacted-AnxA5 membrane proteins expressed in totrophoblasts.

We provided evidence of the role of AnxA5 in trophoblast fusion. However, to our knowledge only syncytin proteins have been characterized to be truly fusogen, suggesting that AnxA5 might play a role in fusion through another step of the process^7^. It is noteworthy that cellular aggregation is considered to be a prerequisite for cell fusion. However, as opposed to other members of the AnxA family (*i.e.* AnxA1 and A2), AnxA5 binds to liposomes without promoting their aggregation^23^. Thus, we proposed that AnxA5 might control trophoblast fusion through interaction with proteins that regulate trophoblast fusion. In search for putative AnxA5-binding proteins in primary human trophoblasts, proteins from subcellular membrane fractionations were separated by SDS-PAGE, blotted to nitrocellulose membranes and subjected to fluorescent ligand blot assays overlaid with AnxA5-CF770 in the absence or presence of an excess of recombinant unconjugated AnxA5 (Fig. 4). Several bands with molecular masses in the range of ~130 kDa to ~15 kDa were detected in the membrane fraction of AnxA5-CF770 ligand blot (Fig. 4). Proteins of corresponding bands that were competed in presence of unconjugated AnxA5, suggesting for putative AnxA5-binding proteins, were isolated from cytotrophoblasts (CT) and syncytiotrophoblasts (ST) membrane fractions and subjected them to size-fractionated SDS-PAGE bands before tryptic digestion to nanoLC-LTQ Orbitrap mass spectrometry analysis. Database searches against proteins in UniProt with Mascot scores >30 identified AnxA proteins (A1, A2, A5 and A6), E-cadherin/catenin complex (β and δ) and ERM proteins (ezrin, radixin and moesin) (Table 1).

### AnxA5 colocalizes with E-Cadherin, β-Catenin and α-Catenin

**Figure 5:**
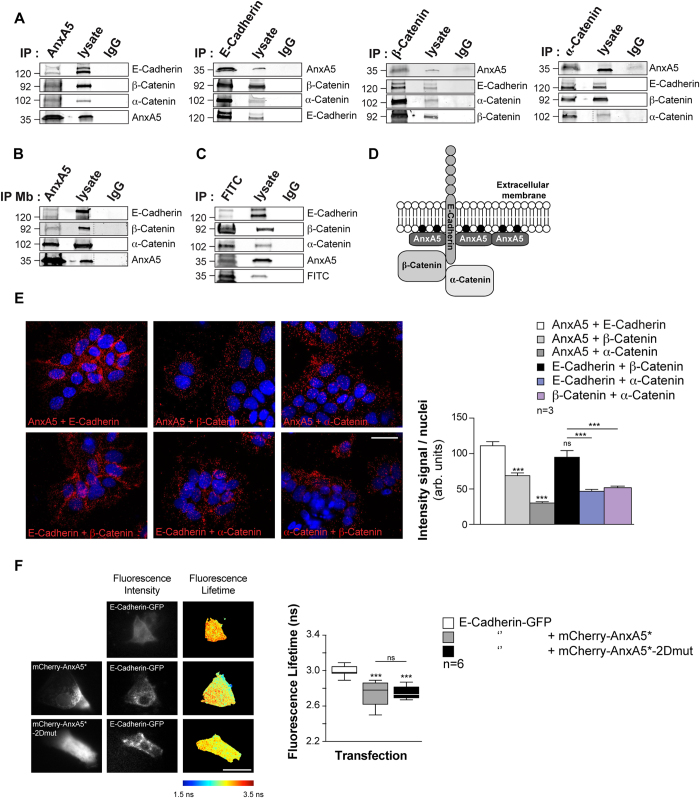
AnxA5 is in the vicinity of E-Cadherin complex. (**A**) Lysates from cytrophoblasts were subjected to immunoprecipitation (IP) with antibodies against AnxA5, E-Cadherin, β-Catenin, α-Catenin and IgG controls. Immunoprecipitates, IgG controls and corresponding lysates were analyzed by immunoblotting for the presence of the indicated proteins. Dotted lines indicate parts combined from a single gel and exposure. (**B**) Lysates from cytrophoblasts membrane fraction were subjected to IP with antibodies against AnxA5 and IgG controls. Immunoprecipitate, IgG control and corresponding lysate were analyzed by immunoblotting for the presence of the indicated proteins. Dotted lines indicate parts combined from a single gel and exposure. (**C**) Trophoblasts were incubated with AnxA5-FITC and corresponding lysates were subjected to IP with antibody against FITC. Immunoprecipitate, IgG control and corresponding lysate were analyse by immunoblotting for the presence of the indicated proteins. (**D**) Illustration of the supramolecular complex including E-Cadherin, AnxA5, β-Catenin and α-Catenin. (•) black circles represent phosphatidylserine (PS). (**E**) Cytotrophoblasts were subjected to PLA (left panel). Cells were stained with pairs of antibodies as indicated: AnxA5-E-Cadherin, AnxA5-β-Catenin, AnxA5-α-Catenin, E-Cadherin-β-Catenin, E-Cadherin-α-Catenin and α-Catenin-β-Catenin. Physical proximity of the molecules was assessed using Duolink technology, generating red spots when molecular proximity was <40 nm and nuclei were stained with DAPI. Scale bar: 15 μm. The intensities of the fluorescent spots generated were normalized to the number of nuclei (right histograms). Quantitative results are expressed as the mean ± SEM of n** = **3 independent experiments (***p < 0.001; ns, non-significant). (**F**) GFP and mCherry fluorescence intensities (left panel) and FLIM (right panel) images of E-Cadherin-GFP-positive cells cotransfected (bottom) or not (top) with mCherry-AnxA5* or mCherry-AnxA5*-2Dmut. The color-coding bar of FLIM images indicates the GFP fluorescence lifetime value. Fluorescence lifetime is presented as a box plot (right panel). Quantitative results are expressed as the mean ± SEM of n** = **6 independent experiments (***p < 0.001) compared with control no FRET (cells transfected with E-Cadherin-GFP alone). Scale bar: 15 μm.

A complex of E-Cadherin and Catenins has previously been shown to regulate trophoblast aggregation and thus cell fusion^5^,^24^. Interestingly, E-Cadherin complex (*i.e.* E-Cadherin, β-Catenin and α-Catenin) have been identified by MS analysis (Fig. 4 and Table 1). For these reasons, we investigated whether AnxA5 and E-Cadherin could be located in close proximity to each other. Interestingly, AnxA5 co-immunoprecipitated with E-Cadherin, β-Catenin and α-Catenin (Fig. 5A). Conversely, immunoprecipitation of E-Cadherin or β-Catenin or α-Catenin pulled down AnxA5 (Fig. 5A). Moreover, immunoprecipitation of AnxA5 from the trophoblast membrane fraction also co-precipitated with E-Cadherin, β-Catenin and α-Catenin (Fig. 5B), whereas none of the interaction partners were co-precipitated with control rabbit or mouse IgG. To further address if the addition of exogenous AnxA5 was functional and reached the E-Cadherin complex, primary human trophoblasts were incubated with AnxA5-FITC and cell lysates were subjected to FITC immunoprecipitation. Interestingly, AnxA5-FITC co-immunoprecipitated with E-Cadherin, β-Catenin and α-Catenin (Fig. 5C). We have recently demonstrated that of the ERM proteins, only ezrin, in complex with the gap junction protein connexin-43, triggers trophoblast fusion^9^. Immunoprecipitations were performed to test the co-localization of AnxA5 with ezrin and AnxA expressed in trophoblasts as previously identified by MS analysis (Fig. 4, Table 1 and Supplementary material Fig. S4A). Interestingly, at the plasma membrane of trophoblasts, AnxA5 did not pull down ezrin, AnxA1, AnxA2 or AnxA6 (Supplementary material Fig. S4B). Our results indicate that AnxA5 appears in a supramolecular complex including E-Cadherin, β-Catenin and α-Catenin at the plasmalemma of human trophoblasts (Fig. 5D).

To examine the proximity of AnxA5 with E-Cadherin, β-Catenin and α-Catenin inside the cell, we next performed proximity ligation assays (PLA, Fig. 5E) on permeabilized cells with pairs of specific antibodies. This demonstrated that AnxA5 was in close proximity (<40 nm) to E-Cadherin, β-Catenin and α-Catenin, as evident from the appearance of red dots (Fig. 5E). Physical proximity was also demonstrated for E-Cadherin with β-Catenin and α-Catenin as well as for β-Catenin and α-Catenin (Fig. 5E). The intensity of red signal and density of dots indicating proximity ligation was high for AnxA5-E-Cadherin and E-Cadherin-β-Catenin, somewhat weaker for AnxA5-β-Catenin and weakest for AnxA5-α-Catenin, E-Cadherin-α-Catenin and β-Catenin-α-Catenin, which might reflect the distance between the interaction partners (Fig. 5E, histograms). Furthermore, PLA was negative when either antibody from each pair was omitted or replaced when nonspecific mouse and rabbit IgG primary antibodies were used (Supplementary material Fig. S4C). In addition, separate immunostaining of each protein revealed localizations that could overlap at the plasma membrane of cytotrophoblasts or syncytiotrophoblasts (Supplementary material Fig. S4D). Finally, *in vivo* interactions between AnxA5 and E-Cadherin were supported by förster resonance energy transfer (FRET)/fluorescence lifetime imaging microscopy (FLIM) experiments in live trophoblasts (Fig. 5F). Trophoblasts were transfected with mammalian expression vectors encoding wild type E-Cadherin fused with GFP alone (E-Cadherin-GFP) or in combination with wild type AnxA5 or AnxA5-2Dmut fused with mCherry (mCherry-AnxA5* and mCherry-AnxA5*-2Dmut respectively) (Fig. 5F). Trophoblasts transfected with both E-Cadherin-GFP and mCherry-AnxA5* or E-Cadherin-GFP and mCherry-AnxA5*-2Dmut showed a significant decrease in the mean GFP fluorescence lifetime. This indicates FRET between AnxA5 or AnxA5-2Dmut and E-Cadherin as compared with trophoblasts that only expressed E-Cadherin (Fig. 5E; 2.74 ± 0.06 ns or 2.75 ± 0.02 ns and 2.99 ± 0.02 ns respectively; p < 0.001). The results of FLIM/FRET analyses suggest that AnxA5 and E-Cadherin interact in trophoblasts directly or in a distance of 10 nm. However, we cannot exclude the existence of an intermediate binding partner *in vivo*. Moreover, this indicates that the AnxA5-2D array is not required for the interaction between E-Cadherin and AnxA5.

### AnxA5 2D-network facilitates trophoblasts aggregation

**Figure 6:**
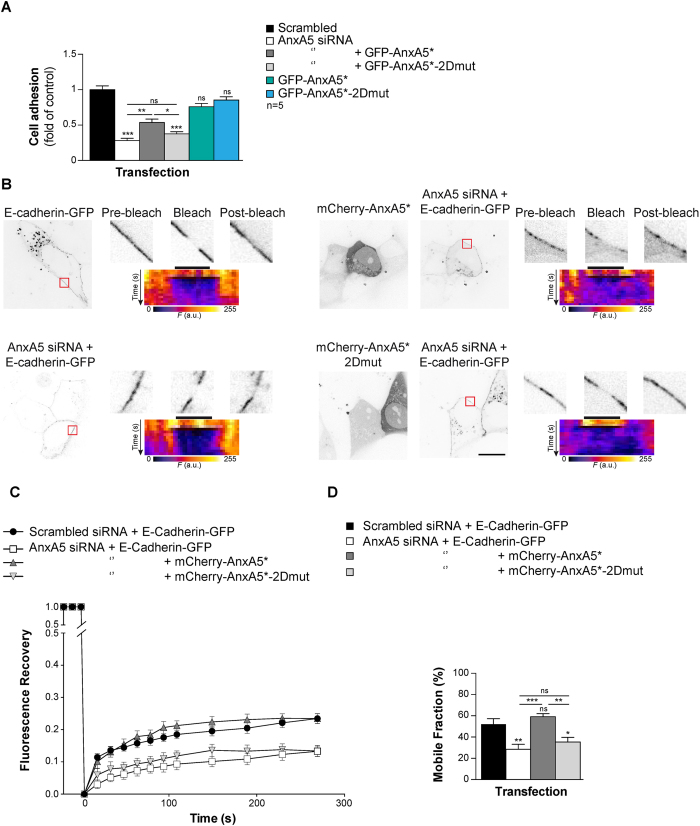
AnxA5 2D-network eases cellular aggregation through E-Cadherin membrane fluidity. (**A**) Trophoblast were transfected with AnxA5 siRNA or scrambled control and reconstituted with GFP-AnxA5* or GFP-AnxA5*-2Dmut and subjected to cell-cell adhesion assay. Quantitative results are expressed as the mean ± SEM. (n** = **5 independent experiments); **p < 0.01; ***p < 0.001; ns, non-significant. (**B**) Trophoblast were co-transfected with AnxA5 siRNA or scrambled control and E-Cadherin-GFP alone or together with mCherry-AnxA5* or mCherryAnxA5*-2Dmut and subjected to Fluorescence Recovery After Photobleaching (FRAP) experiments. GFP and mCherry fluorescence intensity images of E-Cadherin-GFP-positive cells cotransfected (right panel) or not (left panel) with mCherry-AnxA5* (top right) or mCherry-AnxA5*-2Dmut (bottom right). Red squares represent the bleached area. The magnified views of the bleached area show before (pre-bleach), just after (bleach, t = 0 s) and 270 s after photobleaching (post-bleach). Scale bar: 10 μm. The pseudo-color images indicate the corresponding kymogram. The dark bar indicates the bleach area. (**C**) The graph represents the fluorescence recovery after photobleaching versus time measured in 6 independent experiments from different primary cultures, each analysing >10 cells. (**D**) Histograms present the mobile fraction of E-Cadherin-GFP in AnxA5 siRNA or scrambled control transfected cells in presence of absence of co-transfection with mCherry-AnxA5* or mCherry-AnxA5*-2Dmut.

E-Cadherin initiates trophoblast fusion by promoting the aggregation of mononuclear cytotrophoblasts in a calcium dependant manner^5^,^24^. To assess a potential role of AnxA5 in cellular aggregation through its association with E-Cadherin, we performed Vybrant Cell aggregation assays on primary human trophoblasts. Trophoblast aggregation assays performed without Ca^2+^ showed significantly less aggregation than the control (p < 0.001; Supplementary material Fig. S4E). In addition, cytotrophoblasts pre-incubated with a blocking antibody directing the external domain of E-Cadherin reduced cellular aggregation as compared with cells pre-incubated with isotype-matched IgG control (p < 0.001; Supplementary material Fig. S4E). These support for the key role of E-Cadherin in cytotrophoblasts aggregation. Next, cytotrophoblasts were depleted of endogenous AnxA5 by siRNA transfection and reconstituted with either GFP-AnxA5* or GFP-AnxA5*-2Dmut (Fig. 6A). It is noteworthy that silencing and reconstitution experiments did not modify E-Cadherin, β-Catenin and α-Catenin protein levels (Supplementary material Fig. S4F). Finally, we demonstrated by co-immunoprecipitation and PLA that AnxA5 silencing and reconstitution experiments did not affect the composition of the supramolecular complex or its localization at the cell membrane of trophoblasts (Supplementary material Fig. S4G,H). Cells in which endogenous AnxA5 was knocked down presented 70% less cellular aggregation (p < 0.001) compared to cells transfected with scrambled siRNA (Fig. 6A). Interestingly, cell-cell aggregation was partially rescued in cytotrophoblasts transfected with AnxA5 siRNA and GFP-AnxA5* (p < 0.01 compared with AnxA5 siRNA transfected cells). By contrast, cytotrophoblasts reconstituted with GFP-AnxA5*-2Dmut were not rescued for cellular aggregation (approximately 60% less cell-aggregation; p < 0.001 compared with scrambled control). Furthermore, cytotrophoblasts transfected with either GFP-AnxA5* or GFP-AnxA5*-2Dmut displayed the same cell aggregation profile as scrambled siRNA transfected cells (Fig. 6A). All together, this indicates that AnxA5 and the associated 2D-arrays are required for trophoblast aggregation process.

### AnxA5 2D-network is required for E-Cadherin mobility at the plasmalemma

Self-association of AnxA and the resulting network formation impact on the membrane mobility of lipids and proteins^25^,^26^. Noteworthy, the mobility of proteins and lipid fluidity of the plasma membrane affect trophoblast fusion^7^. Our PLA and FLIM/FRET experiments performed in physiological conditions in human trophoblasts, highlighted for a pool of AnxA5 together with E-Cadherin. The spatiotemporal organization of the cellular aggregation machinery including E-Cadherin is crucial to trigger trophoblast fusion^5^,^24^. We therefore, investigated the molecular dynamics of E-Cadherin by Fluorescence Recovery After Photobleaching (FRAP) inside the plasma membrane of primary human trophoblasts. Here, we did not used ionomycin to raise artificially intracellular calcium concentration. This ionophore disrupts cell-cell adhesion, cleaves E-Cadherin, modifies biochemical characteristics of lipids membrane (*i.e.* fluidity and distribution) and enhances PS externalisation, which might interfere with our model of cell fusion^27^,^28^. Thus, the mammalian expression vector encoding for E-Cadherin fused to GFP (E-Cadherin-GFP) was introduced in primary human trophoblasts depleted or not of endogenous AnxA5 by siRNA transfection and rescued with either mCherry-AnxA5* or mCherry-AnxA5*-2Dmut (Fig. 6B–D). The E-Cadherin diffusion coefficient of knockdown and reconstitution experiments ranged from 1–2.5 × 10^−2^ μm^2^ s^−1^, which is consistent with previous work^29^. Cytotrophoblasts transfected with AnxA5 siRNA showed a decrease in E-Cadherin-GFP plasma membrane mobility compared to scrambled control as evident from signal recovery curves (Fig. 6C) and associated mobile fractions (Fig. 6D, approximately 50% less, p < 0.01). However, trophoblasts with AnxA5 knockdown that were reconstituted with mCherry-AnxA5* recovered E-Cadherin-GFP mobility (Fig. 6B–D). By contrast, signal recovery curves and mobile fraction histograms indicated that reconstitution with the AnxA5 variant that does not form 2D-network (mCherry-AnxA5*-2Dmut) did not restore E-Cadherin-GFP mobility (Fig. 6B–D, approximately 30% less mobile fraction compared with scrambled control, p < 0.05). Kymograms (display the temporal evolution of the fluorescent intensity) together with high magnification views and diffusion coefficient value indicated that the fluorescence recovery was associated with E-Cadherin diffusion within the trophoblast membrane, rather than diffusion from cytoplasm to the plasma membrane (Fig. 6B). In summary, our knockdown and reconstitution experiments suggest that AnxA5, through 2D-network formation, plays a role in the regulation of E-cadherin mobility inside the plasma membrane of human trophoblast.

## Discussion

Cells need to complete successive stages to commit syncytial formation^5^. We report that in primary human trophoblast, a pool of AnxA5 facilitates cellular aggregation by self-assembly at the inner membrane of trophoblasts. Through its cellular membrane localization, we show that AnxA5 interacts with the E-Cadherin complex including β-Catenin, α-Catenin and allows E-Cadherin mobility at the plasma membrane subset. We demonstrate that AnxA5 is required for cell-cell aggregation, which is a prerequisite for trophoblast fusion.

*In situ*, we observed high levels of AnxA5 in the villous trophoblast lineage (cyto- and syncytiotrophoblast), which supports a previous study^30^. AnxA5 was found predominantly at the apical pole of syncytiotrophoblast, at the plasma membrane of cytotrophoblasts and particularly at the junction between mononuclear cells and syncytiotrophoblast, where the fusion process occurs. Similarly, we observed *in vitro* AnxA5 at the plasma membrane and at the junction between cytotrophoblasts and/or syncytiotrophoblasts. Moreover, our dual AnxA5 labelling testified for an absence of AnxA5 expression at the outer membrane of aggregated cytotrophoblasts, whereas syncytia displayed AnxA5 externalisation.

By silencing and reconstitution experiments, we established a crucial role of AnxA5 in trophoblast fusion. Interestingly, reconstitution with mutant AnxA5 with impaired ability to self-assemble in 2D-arrays did not restore human trophoblast cell fusion, suggesting that the resulting AnxA5 2D-array controls human trophoblast fusion. It is noteworthy that AnxA5 does not meet all criteria to be defined as a fusogen molecule^2^. Thus, its action in the regulation of trophoblast fusion occurs by interacting with or establishing at the plasma membrane, protein involved in the fusion process. Our proteomic analysis together with our co-immunoprecipitation and PLA assays revealed that a pool of AnxA5 localized at the inner plasma membrane of human trophoblast is in proximity with E-Cadherin, β-Catenin and α-Catenin. Interestingly, these proteins regulate trophoblasts fusion^24^,^31^. Dynamic studies of the relative localization of fuse-tagged AnxA5 and E-Cadherin performed by FLIM FRET assay revealed proximity between these proteins in primary human trophoblasts.

We provided evidence that in culture, recombinant AnxA5 wild type was quickly internalized and similarly to endogenous, reached the E-Cadherin complex to accelerate the fusion process whereas the recombinant 2D-mutant did not. Extracellular AnxA5 endocytosis might occur through macropinocytosis^32^. The amount of exogenous AnxA5 used in our uptake experiments will artificially increase the intracellular concentration and AnxA5 availability, which lead to an acceleration of trophoblast fusion. Our data are in agreement with a recent study showing the important role of AnxA1 and AnxA5 in myoblast fusion^20^, which support for AnxA5 as a key protein regulating fusion processes in various biological contexts. Leikina *et al*. show that extracellular AnxA1 and AnxA5 act in a functionally redundant manner to trigger myoblast fusion^20^. Although in some crucial biological contexts, AnxA proteins might be functionally redundant, we did not identify other members of the AnxA family in the complex formed by AnxA5 at the plasma membrane of human trophoblasts^16^. This indicates that the molecular structure encompassing the E-Cadherin complex in primary human trophoblasts is specific to AnxA5. However, we cannot rule out the involvement of AnxA1 in trophoblast fusion by a different molecular mechanism than AnxA5. Interestingly, AnxA1, A2, A4, A6 and A7, but not A5 aggregate lipid membranes and could therefore putatively trigger cell fusion of competent cells^16^,^23^. This may explain observations done by Leikina *et al*., on extracellular AnxA1-induced myoblast fusion^20^.

E-cadherin allows trophoblast fusion by facilitating efficient cellular aggregation^5^,^24^. Disruption of cellular aggregation by targeting E-Cadherin leads to a defect in syncytialization^24^. Our cellular aggregation assays and FRAP experiments performed with silencing and reconstitution experiments provide evidence that the AnxA5 2D-network is essential to facilitate E-Cadherin mobility inside the plasma membrane and thus trophoblast aggregation. AnxA5 binds to anionic phospholipids such as PS in a Ca^2+^-dependent manner^16^. We propose that following a local increase in intracellular Ca^2+^, AnxA5 binds to PS at the inner-leaflet of the plasma membrane and organizes a 2D-network. This network promotes lateral diffusion of E-Cadherin, which sets up adherens junctions at the plasma membrane and thus facilitates trophoblast fusion. A similar model has been proposed for VE-Cadherin and AnxA2 in maintaining adherens junctions in endothelial cells^33^. We could hypothesis that the entire AnxA5-E-Cadherin complex moves inside the membrane with cytoskeleton interaction to set up at the right time and place E-Cadherin for an efficient trophoblast aggregation and fusion. Alternatively, AnxA5 2D-network is organized on each side of pools of E-Cadherin complex and thus generates mechanical forces and/or modification of membrane fluidity that promotes E-Cadherin mobility. However, further experiments need to be performed to support one of these hypotheses. It has been described that AnxA5 interaction with PS and the formed extended 2D-network reduce the lateral motion of lipid membrane^26^. This discrepancy with our observations in human trophoblasts could be explained by the use in the study of non-physiological models (*i.e.* small unilamellar vesciles or planar supported lipid bilayers). Artificial membranes lack physiological processes that control biochemical properties of the membrane and observations performed with these models might differ from physiological conditions^27^. Interestingly, intracellular Ca^2+^ concentration in subplasmalemma microdomains fits with the lowest concentration of Ca^2+^ that have been described to promote AnxA5 binding to PS and 2D-network assembly^18^,^34^,^35^. Taking into account that Ca^2+^ plays a key role in cell fusion, it would be interesting to decipher the spatiotemporal regulation of the Ca^2+^ homeostasis with the dynamics of AnxA5 anchoring through PS during cell fusion.

An externalization of PS is essential in myoblasts and trophoblasts fusion^7^. In line with these, we provided evidence for PS flip and AnxA5 externalisation in human trophoblasts occurring after the competence and commitment stages either during the fusion pore formation or at the end of the process. Interestingly, this phenomenon was not associated with trophoblasts apoptosis, which is in agreement with similar observations made in myoblast fusion^36^. The purpose and the reason of PS flip occurring in late trophoblast fusion processes remain elusive. In myoblasts, however, PS flip and AnxA5 externalization appear earlier in the fusion process suggesting for differences in fusion behavior depending on cell type^20^.

Anomalies of villous trophoblast differentiation and cell fusion lead to severe placental abnormalities (*i.e*. IUGR and preeclampsia)^14^. Noteworthy, AnxA5 is markedly reduced in the placenta of IUGR and preeclampsia^37^,^38^,^39^. In conclusion, using a physiological model with primary culture of human trophoblasts, we report that AnxA5 forms a 2D-network at the inner-leaflet of the plasma membrane of human trophoblasts. We provide evidence for the involvement of AnxA5 2D-network in coordination of trophoblasts aggregation. The resulting network facilitates the E-cadherin mobility through the trophoblast membrane and thereby controls cell fusion.

## Methods

### Ethical statement

The study was performed according to the Declaration of Helsinki. Placentas were obtained with the patients’ written informed consent. The protocol was approved by the local ethics committee (CCPRB Paris Cochin n° 18–05). Placental tissues were obtained from women aged between 28 and 44 years with uncomplicated pregnancy undergoing normal Cesarean section or legal and voluntary termination of a normal pregnancy at the Cochin Port-Royal and Antony maternity units (Paris, France).

### Cell culture

Villous cytotrophoblasts were isolated from term placentas and cultured as previously described^9^,^40^.

### Immunolocalization studies

Immunocytofluorescence was performed as previously described^41^. Samples were incubated 1 h at 37 °C with AnxA5-A488 (Interchim, France), AnxA5-FITC (5 μg each) or with monoclonal antibody (2.5 μg each) to desmoplakin (Abcam, France); AnxA5 (Sigma-Aldrich, France) or caspase-cleaved CK18 (cCK18; Roche, France) and incubated with the appropriate fluorochrome-conjugated secondary antibody (Alexa Fluor 488 or 555 (1:500, Life Technologies, France)). Differential labelling of cell-surface and total AnxA5 were performed as previously described^42^. To label AnxA5 surface protein, live trophoblasts were incubated with AnxA5 monoclonal antibody (2 μg; Sigma-Aldrich, France) for 30 min at 37 °C. Cells were next fixed with 4% PFA for 15 min at RT and block for 30 min at RT in HBSS with Ca^2+^, 5% BSA and then incubated 1 h with appropriate fluorochrome-conjugated secondary antibody (Alexa Fluor 488 (1:500, Life Technologies, France)). Cells were then post-fix 5 min at RT in HBSS with Ca^2+^, 4% PFA and permeabilized/blocked at RT for 30 min in HBSS with Ca^2+^, 5% BSA, 0.1% Triton X100. Subsequently, to label total AnxA5 protein, cells were incubated ON at 4 °C with AnxA5 polyclonal antibody (2 μg; Abcam, France) in HBSS with Ca^2+^, 5% BSA, 0.1% Triton X100 and incubated with appropriate fluorochrome-conjugated secondary antibody (Alexa Fluor 555 (1:500, Life Technologies, France)). To visualize intracellular AnxA5, extracellular AnxA5 staining was subtracted to the total AnxA5 staining by using ImageJ.

Immunohistochemistry was performed on human first trimester placenta biopsies. Tissue samples were fixed in 4% PFA for 4 h at 4 °C then in 1% PFA at 4 °C ON and next embedded in 4% agarose. Sections (120 μm) were permeabilized with 0.5% triton X-100 and blocked in 10% FFA BSA, 0.01% triton X100 and incubated with anti-AnxA5 (1 μg/ml; Sigma-Aldrich, France) and anti-CK7 (0.9 μg/ml; Dako, Les Ulis, France). Sections were next incubated with the appropriate secondary antibodies as described above.

### AnxA5 binding assay and uptake

Quantification of phosphatidylserine (PS) exposure was performed by annexin A5 binding assay as previously described^43^,^44^. Trophoblasts were incubated in AnxA5 washing buffer: HBSS without Ca^2+^ with 5 mM EGTA for 5 min at RT (control in HBSS with Ca^2+^). EGTA chelates calcium ions that detaches AnxA5 bound to exposed-PS. Subsequently, 5 μg of AnxA5-FITC or AnxA5 Alexa-Fluor-488 conjugated were added to cell culture in AnxA5 binding buffer (culture medium with Ca^2+^) for 1 h at 37 °C and binds to free exposed-PS. Cells were next subjected to cell viability assay or to desmoplakin immunostaining as described above and the fluorescence intensities corresponding to AnxA5-FITC associated with trophoblasts through PS-exposure were quantified using ImageJ. Extracellular AnxA5 uptake was performed in AnxA5 binding buffer for a time course of incubation (1, 3, 6 and 24 h) at 37 °C. Then cells were washed in Anxa5 binding buffer or washing buffer and subjected to desmoplakin immunostaining as described above.

### Cell viability assay

Cell viability was determined by cleaved cytokeratin 18 (cCK18) immunostaining as an early marker for apoptosis according to the manufacturers’ protocols and as previously described^45^. The extent of overlap between cCK18 and AnxA5 staining was quantified by using intensity correlation coefficient-based analysis.

### Protein sample preparation and immunoblot analysis

Total cell extracts were prepared as previously described by^41^. Plasma membrane protein fractions were obtained using the ProteoExtract Native Membrane protein Extraction Kit (Merck Millipore, France) accordingly do the manufacturer’s protocol. Protein samples were resolved by SDS-PAGE and immunoblotted with antibodies to AnxA1, AnxA2, AnxA6 (0.2 μg/ml each, Santa-Cruz Biotechnology, Germany), AnxA5 (2 μg/ml, Sigma-Aldrich, France), α-Catenin (0.5 μg/ml, Life Technologies, France), β-Catenin (0.25 μg/ml, Life Technologies, France), E-Cadherin (0.25 μg/ml, Life Technologies, France), ezrin (0.5 μg/ml, Life Technologies, France), actin (0.8 μg/ml, Sigma-Aldrich, France), or GFP (1 μg/ml, Clontech, France). After incubation with appropriate DyLight Fluor-conjugated secondary antibody (680 or 800 conjugate, Life Technologies, France) blots were revealed by using Odyssey infrared fluorescent system (Li-Cor, France). Conversely, blot with cell membrane factions were incubated with reversible protein stain (Thermo scientific, France).

### Trophoblast fusion assay

Syncytium formation was followed by monitoring the cellular distribution of desmoplakin and nuclei after fixation and immunostaining as previously described^40^,^46^,^47^. Desmoplakin staining at the boundaries of aggregated mononuclear cells gradually disappears during syncytium formation. Cell nuclei were counterstained with DAPI-containing mounting medium. From a random point in the middle of the coverslips, 1000 nuclei contained in desmoplakin-delimited mononuclear cytotrophoblasts and syncytia were counted. Three coverslips were examined for each experimental condition. Results are expressed as the number of nuclei per syncytium. The fusion index was determined as (N − S)/T, where N is the number of nuclei in the syncytia, S is the number of syncytia, and T is the total number of nuclei counted. Trophoblasts were incubated in AnxA5 binding buffer with recombinant AnxA5 or AnxA5-2Dmut or cells were washed at 24, 48 and 72 h of culture in AnxA5 washing buffer and then subjected to fusion assays.

### SiRNA, mammalian expression vectors and transfection

Transfections (of siRNA and plasmids) were performed using Lipofectamine 2000 CD reagent (Life Technologies, France). siRNA transfections [performed as described previously^41^] were performed with stealth AnxA5 siRNA (ANXA5HSS100510, ANXA5HSS100511 or ANXA5HSS100512, Life Technologies, France). ANXA5HSS100510 was selected as the best of the three stealth siRNA. AnxA5 siRNA: 5′-GGGCUGAUGCAGAAACUCUUCGGAA-3′; 5′-CCCGACUACGUCUUUGAGAAGCCUU-3′ and scrambled control (Stealth RNAi siRNA negative control med GC, Life Technologies, France). The transfection efficiency for siRNA into trophoblasts was >80%, as analyzed by adding fluorescein-labeled double-stranded RNA oligomer to parallel cell cultures. Knockdown efficiency was determined by immunoblotting for siRNA targeting AnxA5 protein.

Mammalian vectors (2 μg) were incubated with the cells (in the presence or absence of siRNA) for 24 h at 37 °C. Human AnxA5 mRNA was amplified from total RNA extracted from primary trophoblast by RT-PCR P1(+): 5′-CACCGCACAGGTTCTCAGAGGCA-3′; P1(−): 5′-TTAGTCATCTTCTCCACAGAGCAG-3′ and cloned into pENTR/D-TOPO vector using the Gateway cloning technology (Life Technologies, France). AnxA5-siRNA-insensitive clones (AnxA5*) were generated by introducing three nucleotide switches, G219A, A231G and G240A. AnxA5-siRNA-insensitive-2D arrays mutated (AnxA5*-2Dmut) was generated by mutagenesis with five amino acid substitutions as previously described^22^, R18E, R25E, K29E, K58E and K193E. Inserts containing human AnxA5 wild type, AnxA5* or AnxA5*-2Dmut were transferred from pENTR into pDEST-EGFP or pDEST-mCherry to yield EGFP-tagged or mCherry-tagged fusion protein (EGFP or mCherry tag in N-terminus). In AnxA5-GFP and AnxA5-2Dmut overexpression experiments, cells were washed in AnxA5 washing buffer. E-cadherin-GFP was a generous gift from Dr. Sylvie Dufour and was previously described in ref. 48. Empty destination vector pDEST-mCherry was generously provided by Dr. Terje Johansen and described in ref. 49. All constructs were verified by sequencing. Transfection efficiency was determined to be >45% for all expression vectors.

### Live cell imaging

Kinetic of annexin fusion-tagged protein translocation to plasmalemma was monitor in live cells or fixed trophoblasts using a laser scanning confocal microscope (SP5; Leica) equipped with a 63x N.A. 1.2 water-immersion objective in the line-scan mode. Trophoblasts were incubated with ionomycin (10 μM). For live cells, images were acquired before incubation and automatically within 20 s intervals for 10 min after incubation.

Optical recording of intracellular Ca^2+^ transient was performed by loaded trophoblasts with the membrane-permeant Fluo-4 AM as recommended by the manufacturer protocol. Wide-field images were obtained with Olympus BX50WI upright microscope with a 40 × 0.8 NA water-immersion objective and an ORCA-AG camera (Hamamatsu)^50^. Images were acquired automatically within 60 s intervals. Pseudo-color images display the intracellular Ca^2+^ value coded in hue and the fluorescence intensity coded in intensity.

### Fluorescent ligand blot assay

Plasma membrane protein fractions from cytotrophoblasts or syncytiotrophoblasts were resolved by SDS-PAGE and blots were incubated for 16 h at 4 °C with 0.5 μM of AnxA5-CF770 (Biotium, France) alone or in presence of an excess of unlabeled recombinant AnxA5 (1 μM; Sigma-Aldrich, France). Bound AnxA5-CF770 was visualized using Odyssey infrared fluorescent system (Li-Cor, France).

### Protein identification by LC-MS/MS

Identification of proteins bound to AnxA5-CF770 was performed as described previously^9^.

### Immunoprecipitation

Antibodies (4 μg each) described above (AnxA5, E-Cadherin, α-Catenin and β-Catenin) as well as antibody against the GFP or FITC tag and nonspecific rabbit or mouse IgG (Jackson ImmunoResearch, UK) were covalently coupled to protein G-linked Dynabeads (Life Technologies, France) using BS^3^ (5 mM, Thermo Scientific, France). Cell lysates from cells cultured for 24 h (200 μg of protein) were added to the bead-linked antibodies. Immunocomplexes were immunoblotted with the indicated antibody.

### Duolink^TM^ Proximity Ligation Assay

Interactions between AnxA5, E-Cadherin, α-Catenin and β-Catenin, GFP in trophoblasts were analyzed using the Duolink^TM^ proximity ligation assay according to manufacturer's instructions and as previously described^9^. All controls are presented in Supplementary material Fig. S3F. The quantification of protein proximity was performed by using ImageJ and by normalizing the fluorescence spots generated to the number of nuclei.

### FLIM/FRET experiments

FLIM measurements were done in frequency domain by phase modulation on a custom system based on a commercial module (Lifa, Lambert Instruments, Netherlands) as described previously^51^. The module was attached to aNikonTE2000 TIRF inverted microscope equipped with a Coolsnap HQ CCD camera (Photometrics, USA), a 473 nm modulated laser diode (Omicron, Germany) used for excitation of the donor fluorophore and a 100 × 1.49 NA TIRF objective. The laser light was coupled using an optical fiber to the TIRF illumination arm. Fluorescence emission was selected by a band-pass filter (500–550 nm). Transfected cells were imaged in wide field illumination by a mercury lamp with standard Nikon filter cubes and a Coolsnap Ez CCD camera (Photometrics, USA) prior to FLIM measurements in order to estimate the expression level of both EGFP- and mCherry-tagged proteins. All the experiments were performed at 37 °C with 5% CO2. FLIM images were analyzed on small area containing EGFP- and mCherry-positive structures with the LI-FLIM software (Lambert Instruments, Netherlands) in order to determine the mean fluorescence lifetime for each structure. Time exposure was comprised between 500–1500 ms.

### Cellular aggregation assay

Cellular aggregation assay was performed by adapting the Vybrant Cell Adhesion assay kit (Life Technologies, France). Purified human trophoblasts were plated in both 96 well plate (at density of 1.56 × 10^5^ cells per cm^2^; black plate, clear bottom, Fisher Scientific, France) and in 35 mm cultures dishes (at density of 1 × 10^5^ cells per cm^2^; Fisher Scientific, France). Trophoblasts were co-transfected with AnxA5 siRNA or scrambled control and with GFP-AnxA5* or GFP-AnxA5*-2Dmut. Cells from 35 mm dishes were trypsinized and resuspended either in pre-heated HBSS (control) or in pre-heated AnxA5 washing buffer at a final concentration of 5 × 10^6^ living cells/ml. Cells were next washed in pre-heated HBSS and incubated with 5 μM calcein Red-Orange AM (Life Technologies, France) for 30 min at 37 °C. Subsequently, 5 × 10^4^ living calcein-labeled cells were added to the microplate 96 wells and incubated at 37 °C for 120 min in control buffer without or with nonspecific rabbit IgG (Jackson ImmunoResearch, UK) or E-Cadherin blocking antibody (0.2 mg/ml, Abcam, France). The fluorescence of the microplate was measured before and after a careful wash with HBSS using an EnSpire multimode plate reader (Perkin Elmer, France) settled to an Ex/Em of 577/590 nm. The amount of transfected cells expressing EGFP-tagged protein was monitored to be similar to an Ex/Em of 488/509 nm. The percentage of aggregation was obtained by dividing the corrected fluorescence of adherent cells (background subtracted) by the total corrected fluorescence of cells added to each microplate well.

### Fluorecence Recovery After Photobleaching (FRAP) experiments

For the E-cadherin-GFP with or without mCherry-AnxA5* or mCherry-AnxA5*-2Dmut movies after a 24 h induction of AnxA5 siRNA, cytotrophoblast cultured in IBIDI μ-Slide 8 well (Biovalley, France) were transfected as described above 18 hours prior to observation. The images were acquired on a spinning disk microscope. The Spinning disk microscope is based on a CSU-X1 Yokogawa head mounted on an inverted Ti-E Nikon microscope equipped with a motorized XY Stage. Images were acquired through a 100 × 1.4NA Plan-Apo objective with a QuantEM EMCCD camera (Photometrics, USA). Optical sectioning was achieved using a piezo stage (Nano-z series, Mad City Lab, USA). A Roper/Errol laser bench was equipped with 405, 491 and 561 nm laser diodes, delivering 50 mW each, coupled to the spinning disk head through a single fiber. Multi-dimensional acquisitions were performed in streaming mode using Metamorph 7.7.6 software (Molecular Devices, France). A single region (ROI) of the membrane was photobleached per cell to avoid non-specific bleaching. FRAP analyses on E-cadherin-GFP, thus trophoblasts were maintained in culture medium in IBIDI μ-Slide 8 well and imaged with a FRAP head (Errol and Roper, France), 18 h after transfection. Fluorescence recovery images were monitored by scanning the bleached area (3.2 μm region of the plasma membrane) with an attenuated laser beam (0.9 mW from the pupil of the objective). After photobleaching, images were acquired every 500 ms during 5 s then every 40 s for 270 s. To increase the signal/noise ratio, denoising was proceed by ND-Safir software^52^ and the eventual shift was corrected by Motion 2D^53^. Quantification of the fluorescence recovery was realized by ImageJ software. The intensity of fluorescence was normalized and plotted on the graph using GraphPad Prism 6 (La Jolla, USA).

Kymograms show the FRAP time course. The mobile fraction was determined as (F_∞_ − F_0_)/(F_i_ − F_0_), where F_∞_ is the fluorescence in the bleached region after fluorescence recovery at infinite time, F_i_ is the fluorescence before bleaching and F_0_ is the fluorescence immediately after bleaching. The recovery curves were fit to non-linear regression and the plateau followed by one-phase association equation using GraphPad Prism 6 (La Jolla, USA). Mobile fraction and diffusion coefficient were obtained by fitting curves with GraphPad Prism 6 (La Jolla, USA).

### Statistics

Quantitative data are presented as mean ± SEM. Statistical differences between groups were evaluated by using Student’s unpaired *t*-test or ANOVA [post hoc analysis (Tukey)], as appropriate.

## Additional Information

**How to cite this article**: Degrelle, S. A. *et al*. Annexin-A5 organized in 2D-network at the plasmalemma eases human trophoblast fusion. *Sci. Rep.* **7**, 42173; doi: 10.1038/srep42173 (2017).

**Publisher's note:** Springer Nature remains neutral with regard to jurisdictional claims in published maps and institutional affiliations.

## Supplementary Material

Supplementary Material

## Figures and Tables

**Table 1:** Identification of AnxA5-binding proteins.

Protein	UniProt. Acc. No.	Mass (Da)	Mascot Score	Coverage (%)	No. of peptides
Annexin A1	P04083	38690	766	50.9	15
Annexin A2	P07355	38580	2018	67.8	28
Annexin A5	P08758	35914	555	47.5	13
Annexin A6	P08133	75826	469	26.3	14
E-Cadherin	P12830	97396	145	3.7	3
β-Catenin	P35222	85442	280	15	10
δ-Catenin	O60716	108103	95	4	3
Ezrin	P15311	69370	1295	50.5	32
Radixin	P35241	68521	666	30.2	17
Moesin	P26038	67778	466	15.1	11

Interacted-AnxA5 proteins from trophoblast membrane fraction were identified by nanoLC-LTQ Orbitrap mass spectrometry analysis of tryptic digests of bands excised from polyacrylamide gels after SDS-PAGE. Acc No, accession number; MW, molecular weight.
