# Supplementary material for: Annexin-A5 organized in 2D-network at the plasmalemma eases human trophoblast fusion
Source: Sci Rep. 2017 Feb 8;7:42173. doi: 10.1038/srep42173 (PMC5297248; doi:10.1038/srep42173)

SUPPLEMENTARY MATERIAL TO:

Annexin-A5 organized in 2-dimensional network at the plasmalemma eases human trophoblast fusion

by

Severine Degrelle, Pascale Gerbaud, Ludovic Leconte, Fatima Ferreira & Guillaume Pidoux

Legends to Supplementary Figures

Supplementary Figure S1: AnxA5 controls trophoblast fusion in a dose dependent manner. (**A**) Immunoblot analysis of AnxA5 in human trophoblast (26 µg) and of recombinant AnxA5. (**B**) As controls for experiments in Figure 1A, human placental biopsies were immunostained with isotype-matched IgG controls (mouse IgG and rabbit IgG) and nuclei were counterstained with TOPRO-3. Scale bar: 10 µm. (**C**) After 24 or 72 h in culture, Trophoblasts were separately co-immunostained with isotype-matched IgG controls. Nuclei were counterstained with DAPI (blue). Scale bar: 15 µm. (**D**) Trophoblasts were incubated with increasing concentrations of recombinant AnxA5 and immunostained for desmoplakin (DSP, green). Nuclei were counterstained with DAPI (upper panel). Effects of recombinant AnxA5 on cell fusion were plotted as the remaining mononuclear cells and fusion indices (lower panel). Scale bar: 15 µm. (**E**) Trophoblasts were washed in presence or absence of EGTA and immunostained for desmoplakin (DSP, red). Nuclei were counterstained with DAPI (blue). Effects of EGTA wash on cell fusion were plotted as the remaining mononuclear cells and fusion indices. Scale bar: 15 µm. Results are expressed as the mean ± SEM of n = 3 independent experiments (ns for non-significant, *** $p < 0.001$).

Supplementary Figure S2: AnxA5 promotes trophoblast fusion without apoptosis. (**A**) Cell viability was quantified during trophoblast fusion. Human trophoblasts were left untreated or treated with EGTA before incubation with AnxA5-FITC. Histograms represent the viability (%) of trophoblasts quantified by cleaved cytokeratin 18 (cCK18) immunostaining after 24 h and 72 h in culture. (**B**) Viability of trophoblasts induced for cell fusion with recombinant AnxA5 (10 µg) was quantified as in (A). (**C**) Trophoblasts in culture were treated similarly as described in (A) and (B) and were next immunostained for cCK18

and nuclei were counterstained with DAPI (blue). Correlation analysis of co-staining between AnxA5-FITC (green) and cCK18 (red) are presented. Scatter plots show pixels where the intensity of a given pixel in the green and red images corresponds to the x- and y-coordinate, respectively. Pearson's correlation coefficients (R) of overlaps are indicated. (**D**) Trophoblasts were induced for fusion with recombinant AnxA5 (10 µg) and the correlation of co-staining between AnxA5-FITC and cCK18 was performed as in (C). Scale bar: 15 µm. Results are expressed as the mean ± SEM of n = 3 independent experiments (ns for non-significant, *** p < 0.001).

Supplementary Figure S3: AnxA5 controls human trophoblast fusion

(**A**) Immunoblot analysis of AnxA5 and actin levels in trophoblasts transfected with specific AnxA5 siRNA or scrambled controls at 72 h of culture (upper-left panel). Level of AnxA5 was assessed by densitometric scanning of immunoblots and normalized to actin levels in the same blots (upper-right panel). (**B**) Trophoblasts transfected with AnxA5 siRNA or scrambled control alone and simultaneously cultured with recombinant AnxA5 were stained for desmoplakin (DSP, green) and nuclei (DAPI, lower-left panel). Scale bar: 15 µm. Corresponding fusion indices are presented in histograms (right panel). Quantitative results are expressed as the mean ± SEM. (n=3 independent experiments); * $p < 0.05$; ** $p < 0.01$; *** $p < 0.001$; ns, non-significant compared with scrambled control. (**C**) Trophoblasts were transfected with GFP-AnxA5* or GFP-AnxA5*-2Dmut and incubated with or without ionomycin (10 µM) for 270 s at 37°C. Subcellular localization of constructs were monitored by confocal microscopy. Yellow arrowheads indicated relocations of GFP-AnxA5* and GFP-AnxA5*-2Dmut after treatment with ionomycin. Scale bar: 15 µm. (**D**) Similarly to (C) trophoblasts were transfected with mCherry-AnxA5* or mCherry-AnxA5*-2Dmut and treated with ionomycin (10 µM) for 270 s at 37°C. Pictures (left panel) show the subcellular localization of constructs monitored by confocal microscopy. Yellow arrowheads indicated relocations of mCherry-AnxA5* and mCherry-AnxA5*-2Dmut after treatment with ionomycin. Scale bar: 2 µm. Graph (right panel) represents the corresponding relative fluorescence intensity of cytoplasmic mCherry-AnxA5* and mCherry-AnxA5*-2Dmut over time. (**E**) Viable, primary human trophoblasts were loaded with Fluo4-AM to monitor the kinetics (over 60 min) of spontaneous calcium waves. Cell boundaries are indicated with dashed lines. White squares indicate the region of interest (ROIs) from corresponding cells subjected to kymograph analysis (right panel). Intensity of fluorescence corresponding to

intracellular calcium was mapped to pseudocolors as indicated by the color-scale [*F*, in arbitrary units (a.u.)]. Scale bar: 15 μm.

Supplementary Figure S4: AnxA5 localizes at the cellular membrane with E-Cadherin, α-Catenin, β-Catenin, but not with ezrin or other AnxA proteins. (**A**) Immunoblot analysis of AnxA proteins family in trophoblast after 24 h of culture. (**B**) Lysates from cytotrophoblasts membrane fractions were subjected to immunoprecipitation (IP) with antibodies against AnxA5 and isotype-matched IgG controls. Immunoprecipitates, IgG controls and corresponding lysates were analysed by immunoblotting for the presence of indicated proteins. (**C**) As controls for experiments in Figure 5D, trophoblasts were subjected to proximity ligation *in vitro* assay using Duolink technology. Trophoblasts were stained with pairs of antibodies or individual antibody alone as depicted in the figure: mouse IgG (mIgG)-rabbit IgG (rIgG), AnxA5, E-Cadherin, α-Catenin, β-Catenin and nuclei were counterstained with DAPI. Scale bar: 15 μm. (**D**) Trophoblasts in culture were co-immunostained for desmoplakin (DSP) and E-Cadherin or α-Catenin or β-Catenin and nuclei were counterstained with DAPI. Scale bar: 15 μm. (**E**) Trophoblasts adhesion assay were performed in presence (control) or absence of Ca^{2+} (wo Ca^{2+}) or incubated with IgG control or with a blocking antibody directed against the extracellular domain of E-Cadherin (+ E-Cadherin Ab). (**F**) Trophoblast were transfected with AnxA5 siRNA or scrambled control alone or together with mammalian expression vectors directing the expression of siRNA-resistant GFP-AnxA5 (GFP-AnxA5*) or GFP-AnxA5 with mutations in 2D-array organization sites (GFP-AnxA5*-2Dmut) and subjected to immunoblot analysis with indicated antibodies. (**G**) Cells transfected as in E were subjected to immunoprecipitation (IP) of GFP. Immunoprecipitates and corresponding lysates were analyzed by immunoblotting for the presence of the indicated proteins. (**H**) Cells were next stained with a pair of antibodies to AnxA5 or GFP and subjected to proximity ligation *in vitro* assay (PLA). The interaction of molecules stained with the pairs of antibodies was then assessed using Duolink technology. Red dots show molecular proximity (< 40 nm). Nuclei were counterstained with DAPI (left panel). Histograms represent the number of the red dot signals normalized by the number of nuclei. Scale bar: 15 μm. Results are expressed as the mean ± SEM of n=3 independent experiments. (*** $p < 0.001$).

Degrelle et al, Supplementary Figure S1

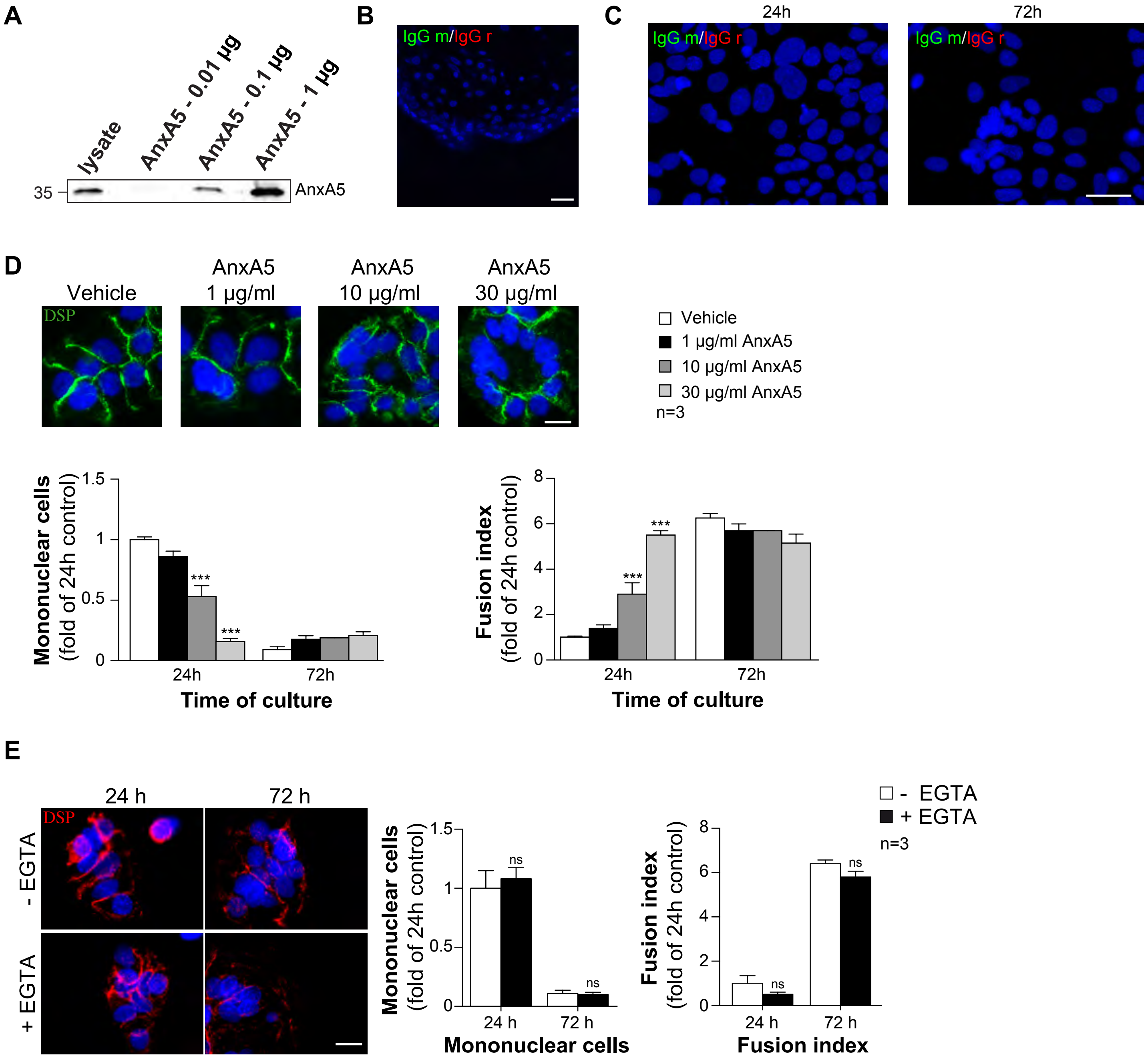

Degrelle et al, Supplementary Figure S2

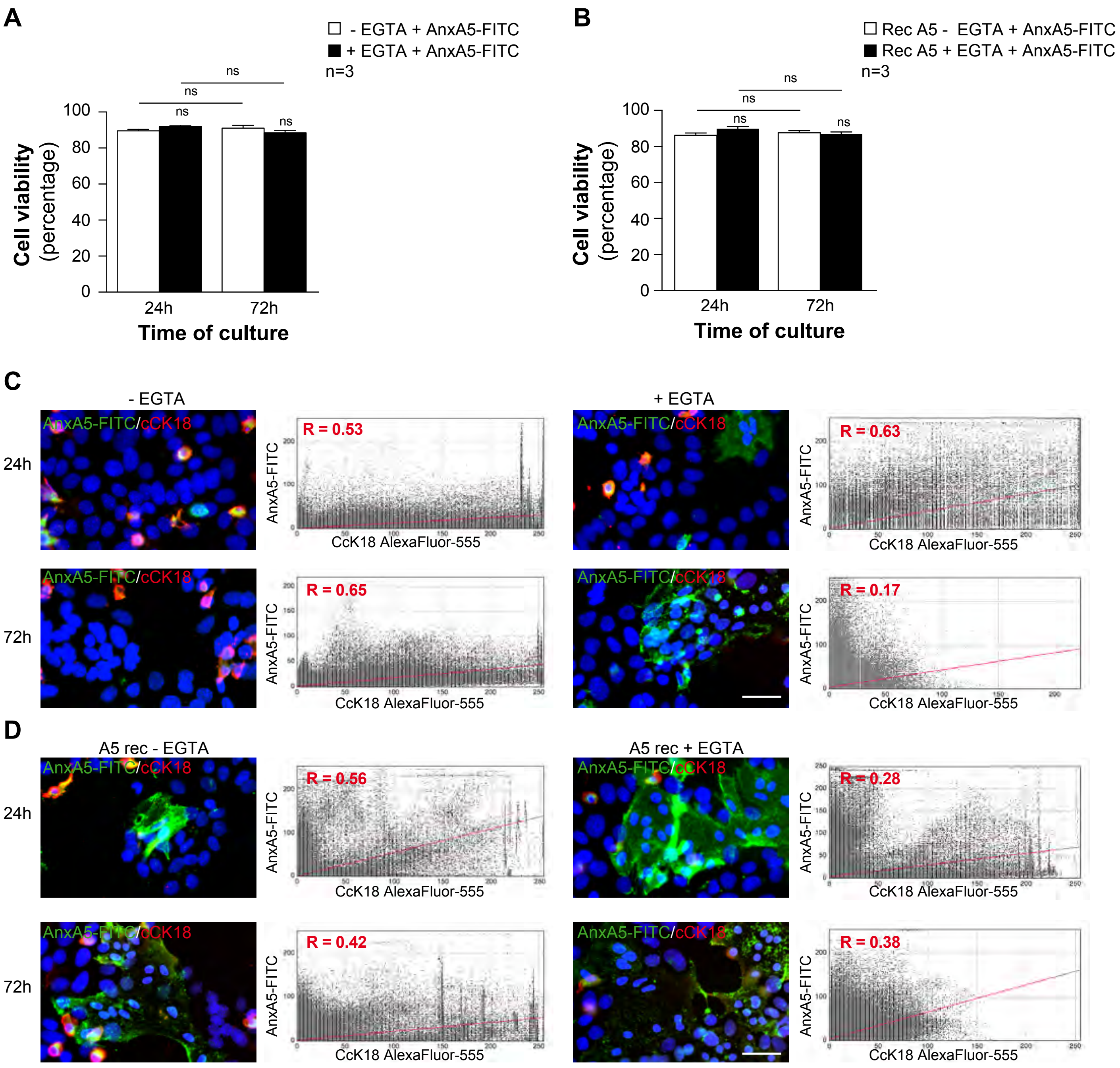

Degrelle et al, Supplementary Figure S3

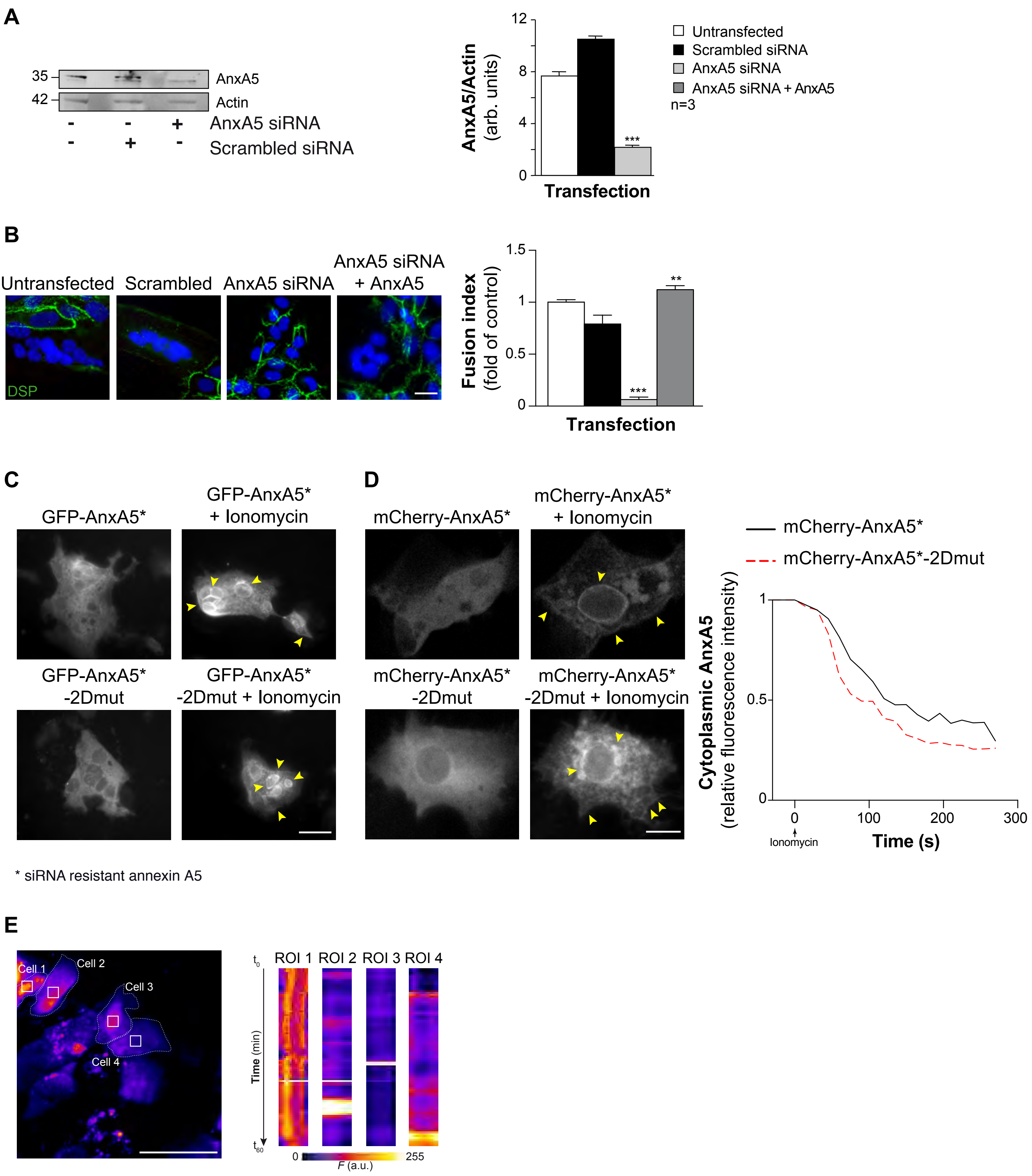

Degrelle et al, Supplementary Figure S4

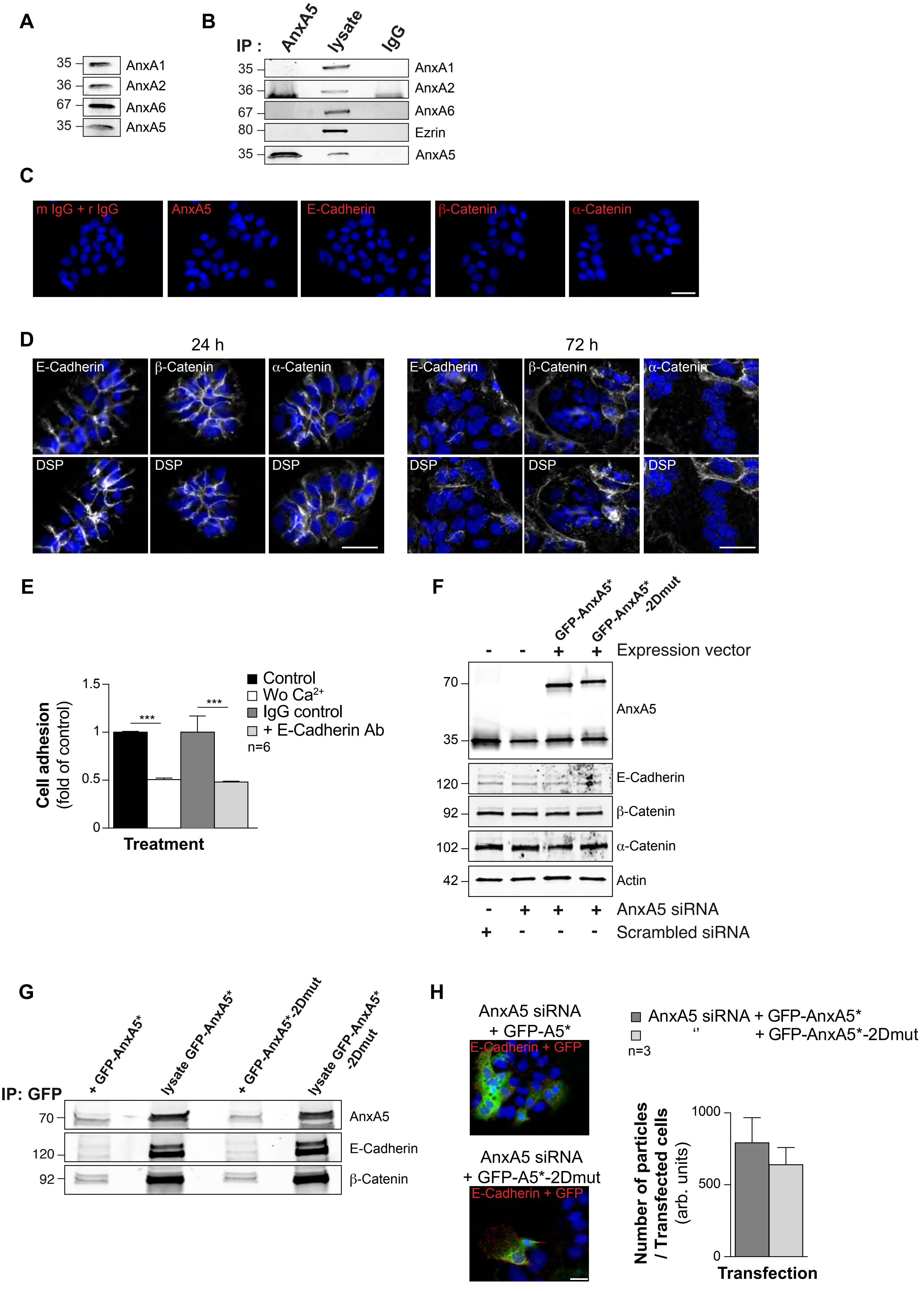

Supplement: Supplementary Material [file srep42173-s1.pdf]
